# A Long Survival Woman with Primary Small-Cell Carcinoma of the Gallbladder: Role of Chemotherapy Maintenance

**DOI:** 10.7759/cureus.1368

**Published:** 2017-06-19

**Authors:** Choukri Elm’hadi, Meryem Zerrik, Hassan Errihani, Mohammed Ichou

**Affiliations:** 1 Department of Oncology, National Institute of Oncology Sidi Mohamed Ben Abdellah, Rabat, Morocco; 2 Department of Internal Medicine, Mohammed V Military Teaching Hospital of Rabat, Morocco; 3 Department of Oncology, Mohammed V Military Teaching Hospital of Rabat, Morocco

**Keywords:** small-cell carcinoma, gallbladder, metastatic, maintenance, chemotherapy

## Abstract

Gallbladder small-cell carcinoma (SCC) is an extremely rare cancer characterized by early metastases and associated with poor survival outcomes. The therapeutic options are limited in this indication and dedicated prospective trials are difficult to achieve.

Maintenance chemotherapy is an evolving concept in medical oncology whose goal is to prolong chemotherapy-induced response. The role of maintenance therapy has been demonstrated especially in many cancers but the results remain controversial in small cell cancer.

We report a case of a 49-year-old woman admitted in our institution with biliary colic, postprandial bilious vomiting, right hypochondrial mass, and deteriorated general condition. Abdominal computed tomography (CT) revealed a mass of the gallbladder with lymphadenopathy in the hepatic hilum and multiple liver metastases. CT-guided biopsy was performed that showed small, round cells with high nuclear-to-cytoplasmic ratio, and frequent atypical mitosis, which is consistent with high-grade small cell neuroendocrine carcinoma. Tumor cells were positive for chromogranin A, synaptophysin, and CD56. Ki-67 shows a high proliferation rate with 90% tumor cell staining and the diagnosis of gallbladder SCC was confirmed. The treatment used a combination of carboplatin and etoposide, interrupted by the generalized discomfort and shortness of breath during the second course of the etoposide. Repeated CT scan showed a partial radiological response in the order of 35% and carboplatin monotherapy was maintained with good tolerance and stability of the disease until the 11th cure. Thrombocytopenia at 70,000 per mm^3^ appeared, and its persistence forced the cessation of this treatment. Five months later, the disease progressed and second-line chemotherapy by irinotecan was given weekly. The death occurred 18 months after initiation of medical treatment due to hepatocellular insufficiency. Maintenance therapy in SCC should be considered as a promising therapeutic option when it is well tolerated.

## Introduction

Gallbladder small-cell carcinoma (SCC) is an extremely rare malignancy that was first described in 1981 [1]. This aggressive tumor is characterized by early metastases and is associated with poor survival outcomes [1]. Median survival was 13 months for all patients and nine months for patients with metastatic disease [1]. So, the therapeutic options are limited in this indication and dedicated prospective trials are difficult to achieve [1-2].

Maintenance chemotherapy is an evolving concept in medical oncology aiming to prolong the response induced by induction chemotherapy and to slow the progression of an oncological disease considered as non-curable [3]. The role of maintenance therapy has been demonstrated especially in non-small-cell lung cancer and colon cancer [4].

We report a case of a 49-year-old woman with a metastatic gallbladder SCC to the liver, having survived long after maintenance chemotherapy.

## Case presentation

**Figure 1:**
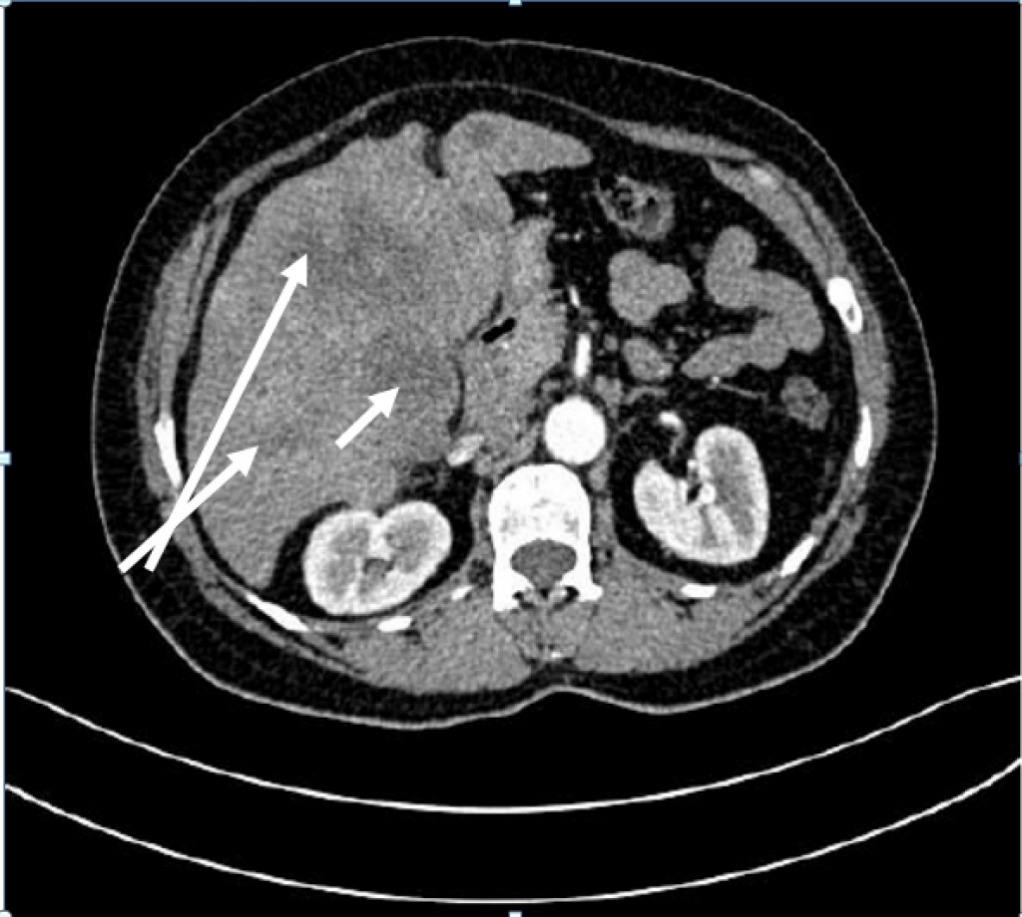
Abdominal computed tomography: 10.5 × 9 cm mass replacing the gallbladder with liver metastases.

A 49-year-old woman, without past medical or surgical history, was admitted in our institution with digestive symptoms such as biliary colic, postprandial bilious vomiting, right hypochondrial mass, and a deteriorated general condition. Physical examination revealed an enlarged gallbladder and hepatomegaly. Routine blood chemistry showed total bilirubin at 12 mg/dl, direct bilirubin at 4 mg/dl, aspartate aminotransferase (AST) at 51 U/l, alanine aminotransferase (ALT) at 26 U/l, alkaline phosphatase at 250 U/L, and gamma glutamyl transpeptidase at 325 U/L. Complete blood count was normal and we did not check tumor markers. The ultrasonography revealed a 10.5 × 9 cm mass replacing the gallbladder with lymphadenopathy in the hepatic hilum. The mass caused compression to adjacent structures such as the liver, mesenteric fat, and maybe the second duodenal portion. Multiple metastases involving the liver were also observed. Abdominal computed tomography (CT) confirmed the above findings (Figure 1).

**Figure 2:**
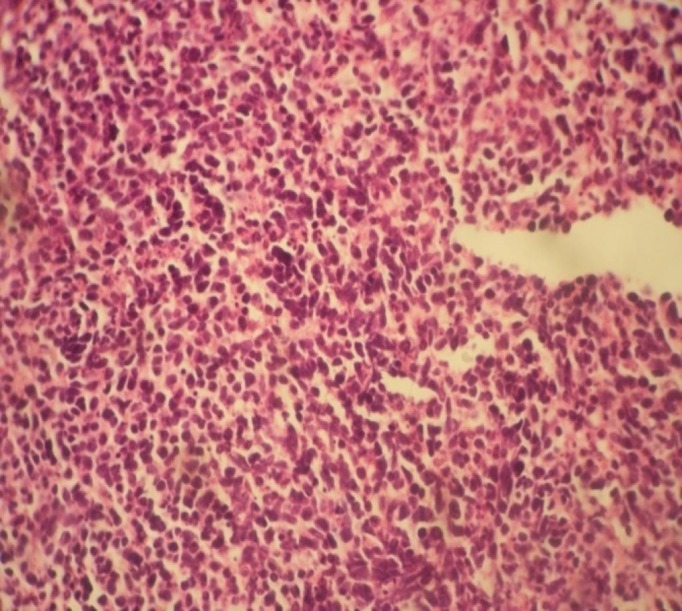
Monotonous proliferation of small cells with round basophilic nuclei and scanty cytoplasm resembling neuroendocrine carcinoma (hematoxylin and eosin staining x100).

**Figure 3:**
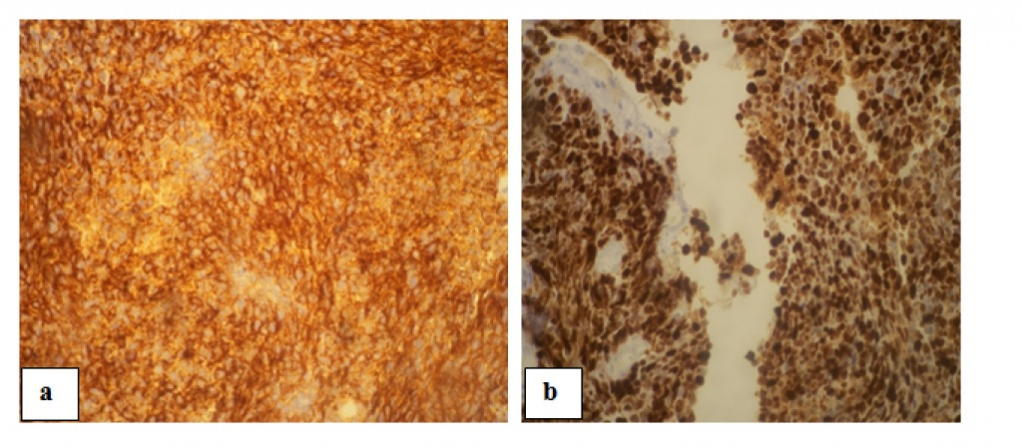
Membrane staining with: a) chromogranin b) synaptophysin (x200).

A performed CT-guided biopsy showed small, round cells with high nuclear-to-cytoplasmic ratio, and frequent atypical mitosis, which is consistent with high-grade small cell neuroendocrine carcinoma (Figure 2). Tumor cells were positive for chromogranin A, synaptophysin (Figure 3) and CD56 (cluster of designation 56), and negative for cytokeratin 7, 20 and anti-hepatocyte. Ki-67 (Ki-67 index) shows a high proliferation rate with 90% tumor cell staining and the diagnosis of an SCC of the gallbladder was confirmed.

Performed CT scans of the brain, chest, pelvis, and bone were negative for any other metastatic disease and no primary lung mass was found. Due to the multiple liver metastases, palliative chemotherapy was indicated. Because of poor performance status, our patient was considered unfit for cisplatin. She was treated with carboplatin (area under the concentration-time curve [5]) and etoposide 100 mg/m² at day 1, day 2, and day 3.

**Figure 4:**
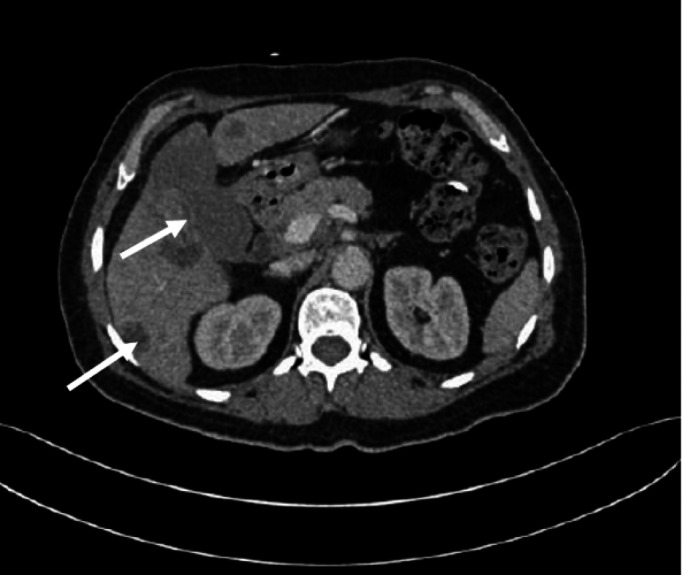
Abdominal computed tomography showed partial response after two cycles of chemotherapy.

She received two cycles of chemotherapy, interrupted by the generalized discomfort and shortness of breath during the second course of the etoposide. Repeated CT scan showed a partial radiological response in the order of 35% according to the RECIST (Response Evaluation Criteria in Solid Tumor) 1.1 criteria (Figure 4). The multidisciplinary consultation meeting retained the maintenance of carboplatin alone until progression or intolerance. Subsequent reassessments have shown a stable disease with good tolerance of treatment after the fifth cure, eighth cure and 11th cure. Then, thrombocytopenia at 70,000 per mm^3^ appeared, and its persistence forced the cessation of this treatment. A therapeutic break is offered to the patient. Five months later, the disease progressed with the appearance of bone metastases and new hepatic metastases. The patient received second-line chemotherapy by irinotecan weekly at the dose of 100 mg/m²/day. The evolution was rapidly progressive by the appearance of jaundice which quickly complicated by hepatocellular insufficiency. The death occurred 18 months after the start of medical treatment.

## Discussion

SCC of the gallbladder represents 0.5% of all gallbladder cancers. These poorly differentiated tumors resemble their more common counterparts arising in the bronchopulmonary system. They exhibit similar histological features and aggressive behavior with a high propensity for invasive growth, and early lymphatic nodes and distant metastases, and have an exceedingly poor prognosis [5].

There have been numerous cases and small series reported of this pathology in the literature. It is a disease that most commonly affects an elderly, female population [1]. Similar to adenocarcinoma, there is a strong association between cholelithiasis and this tumor. Interestingly, there are no neuroectodermal cells in the gallbladder mucosa [5-6]. This had led investigators to postulate that the SCC arises from metaplastic epithelium of the gallbladder wall [6]. This hypothesis is supported by the presence of metaplasia in the gallbladder with chronic cholecystitis [6].

The clinical behavior of this disease includes an increased incidence in the elderly population with median age of 67 years, female preponderance, late discovery in the disease process when adjacent organ systems have been violated or the biliary system becomes obstructed, and frequency of metastases as 75% of the time to the lymph nodes (88%), liver (88%), lung (23%), and peritoneum [2-6]. The presentation generally consists of non-specific symptoms including upper abdominal pain, jaundice, mass effect, bleeding, and weight loss.

Based on the clinicopathological similarity, treatment used for small cell lung carcinoma has also been employed for extrapulmonary small cell cancer [7]. For first-line chemotherapy for metastatic disease, platinum-based combination regimens with etoposide in western countries, or irinotecan in Japanese patients have often been prescribed. Salvage-line chemotherapy is chosen based on the platinum-free interval and might include the original regimen, topotecan monotherapy, or amrubicin for Japanese population [7].

Due to the low incidence of gallbladder SCC, we could not give unequivocal directions on treatments including specific chemotherapy regimens [1]. Published treatment regimens are highly variable and chemotherapeutic agents of choice are cisplatin, etoposide, and 5-FU [2].

Even after robust responses to initial chemotherapy, small-cell cancer recurs easily due to the emergence of drug-resistant cells [8]. Therapeutic approaches have been evaluated to improve outcomes by dose-intensification strategies or by adding antiangiogenetic agents to the standard chemotherapy, but none of these approaches have demonstrated reproducible benefits over standard platinum-based therapy [8]. A large number of molecular targeted drugs and immunomodulators are currently in clinical development.

Maintenance therapy is an old concept that has shown an obvious benefit on survival in many chemosensitive cancers [4]. Maintenance or consolidation treatments in extensive-stage small-cell lung cancer have been extensively studied with contrasting results. A survival advantage is suggested for maintenance chemotherapy and interferon-alpha, but its clinical impact needs to be confirmed by further studies [9]. Also, maintenance sunitinib of 37.5 mg/day following induction chemotherapy with platinum/etoposide improves median progression-free survival by 1.6 months. This tyrosine kinase inhibitor has shown its safety and should be further evaluated in phase III studies [10].

In our case, the patient had a metastatic SCC of the gallbladder, which was in contrast to the natural history of SCC with a low response rate and prolonged survival beyond one year. The maintenance of cytotoxic pressure could explain this benefit by delaying the emergence of resistant clones.

## Conclusions

On the basis of literature findings, gallbladder SCC is an extremely rare cancer characterized by early metastases and associated with poor survival outcomes. The therapeutic options are limited due to the difficulty in achieving clinical trials dedicated to this orphan neuroendocrine carcinoma. The present topic is a very rare case about a woman with a histologically confirmed metastatic SCC of the gallbladder, successfully managed by systemic administration of carboplatin and etoposide combination that induced a shorter but lasting response thanks to the maintenance chemotherapy by carboplatin alone. This old concept of maintenance having proved its effectiveness in many cancers has been extensively studied in SCC with contrasting results. Gastrointestinal oncology community should consider maintenance therapy as a promising therapeutic option when it is well tolerated.
